# Discrepancy between self-reported and observed hand hygiene behavior in nurses and physicians

**DOI:** 10.1186/1753-6561-5-S6-P120

**Published:** 2011-06-29

**Authors:** T Watanabe

**Affiliations:** 1 Department of Infection Control, Okayama University Hospital, Okayama, Japan

## Introduction / objectives

The most effective method to promote health care workers’Â Hand Hygiene (HH)Â is observation and combined with an investigation of perceptions concerning HH further increases effectiveness.

## Methods

Data were collected at a university hospital in Japan. Nurses and physicians’ perceptions regarding their own HH adherence were measured using an anonymous questionnaire based on the World Health Organization’s (WHO) “Five Moments for Hand Hygiene.” The respondents were asked about their HH adherence in each of the five situations, and the responses were made using a 5-point scale (0%, 25%, 50%, 75%, and 100%). For the analysis, these data were converted into points (0, 1, 2, 3, and 4, respectively).Â Observations were performed in 17 ordinal wards and 4 intensive care units.

## Results

A total of 137 questionnaires were returned from 126 nurses and 11 physicians. The nurses' mean self-reported HH adherence scores for 1) before touching a patient, 2)after touching a patient, 3) after touching a patient’s surroundings, 4) before an aseptic/clean procedure, and 5) after a risk of body fluid exposure were 2.59, 3.02, 2.25, 3.63, and 3.72, respectively, while the physicians’ mean scores were 3.27, 3.45, 2.64, 3.91, and 3.91, respectively. The observed nurses’ adherences were 62.0%, 72.8%, 43.0%, 70.8%, and 87.4%, respectively, while the physicians’ adherences were 25.4%, 52.8%, 42.9%, 34.8%, and 96.5%, respectively.Â Seventy-three percent of the HH failures among physicians before the performance of an aseptic/clean procedure were due to glove use.

## Conclusion

The self-reported HH adherence and the observed HH adherence were measured for nurses and physicians.Â The observed adherence before an aseptic/clean procedure was lower than the self-reported adherence, especially among physicians. This was due to glove use.

## Disclosure of interest

None declared.

